# Chronic Cadmium Exposure and Genetic Polymorphisms of *MMP-2* and *MMP-9* in a Population Exposed to Steel Slag in the State of Rio de Janeiro, Brazil: A Cross-Sectional Study

**DOI:** 10.3390/ijerph192215304

**Published:** 2022-11-19

**Authors:** Jamila A. Perini, Mayara C. da Silva, Lorena V. Correa, Yasmin M. Silva, Renato M. Borges, Maria de Fátima R. Moreira

**Affiliations:** 1 Laboratório de Pesquisa de Ciências Farmacêuticas—LAPESF, Programa de Pós-Graduação em Ciência e Tecnologia Ambiental, Universidade do Estado do Rio de Janeiro (UERJ), Av. Manuel Caldeira de Alvarenga, 1.203, Rio de Janeiro 23070-200, RJ, Brazil; 2 Escola Nacional de Saúde Pública Sergio Arouca, Fundação Oswaldo Cruz (ENSP/Fiocruz), Rio de Janeiro 21041-210, RJ, Brazil

**Keywords:** cadmium exposure, *MMP-2*, *MMP-9*, genetic polymorphism, environmental health, heavy metal

## Abstract

Genetic polymorphisms in the matrix metalloproteinases (*MMPs*) family genes may be associated with cadmium (Cd) levels and its adverse effects. This study investigated the impact of *MMP-2* and *MMP-9* polymorphisms on Cd levels in 238 residents of a condominium in Rio de Janeiro, Brazil, built over an industrial steel slag waste. Polymorphisms were genotyped using TaqMan validated assays, and the Cd levels were measured in blood (BCd) and urine (UCd) samples by atomic absorption spectrometry. Associations were evaluated by linear correlation coefficients and multiple logistic regression, using odds ratios (OR) and 95% confidence intervals (CI). Mean age was 50 ± 15 years; 58% were female, 69% non-smokers. Mean concentrations for BCd and UCd were 0.70 ± 0.2 μg L^−1^ and 0.56 ± 0.55 μg L^−1^, respectively. Smoking status was associated with BCd ≥ 0.70 μg L^−1^ (OR = 2.9; 95% CI = 1.6–5.9). *MMP-9 rs17576 A > G* was associated with BCd ≥ 0.70 μg L^−1^ (OR = 2.11; 95% CI = 1.10–4.05) and UCd ≥ 0.56 μg L^−1^ (OR = 3.38; 95% CI = 1.82–7.65). Knowing possible individual predisposing factors is essential to understand Cd toxicity, and to improve the monitoring of high-risk populations.

## 1. Introduction

Cadmium (Cd) is widely used in industrial activities such as ore refining, smelting, metal plating and coating, and battery production, and is improperly released into the environment as particulate matter and fumes due to its volatilization capacity [1]. The solid waste generated in the process of steelmaking contains a wide variety of hazardous chemicals, including Cd and other metals, which can undergo toxicological interactions, triggering different effects from those induced by Cd exposure alone and posing a risk to the environment and the health of populations living nearby [2,3]. Cd is a toxic metal, and this exposure is a global public health concern that has been shown to exert acute health effects on kidneys, bones, and the nervous, cardiovascular, and respiratory systems. Additionally, Cd and its compounds are classified by the IARC as a human carcinogen [4,5,6,7]. Exposure to Cd may occur through air pollution, occupational activities, smoking, and contaminated food and soil [8,9].

The risk of exposure to metals in soil contaminated by steel industry waste has been previously described for the population in the present study, which resides in a condominium built over the land used by a specific industry as a steel slag disposal in the city of Volta Redonda, Rio de Janeiro, Brazil. It was built between 2000 and 2001, on the east side of the municipality, between the right bank of the Paraíba do Sul river and the Lúcio Meira highway (BR-393). The used land was donated by the steel company to the metalworkers’ union and with funding from Caixa Econômica Federal, a state-owned Brazilian financial services company. To prevent the population from having direct contact with the ground, all sidewalks, streets, backyards, blocks, and squares were waterproofed. In addition, the area has restrictions on the use of soil and groundwater and the residents use treated water from the municipality’s Water and Sewerage Company. However, remediation measures have not yet been carried out and exposure occurs through the inhalation of dust from the tailings pile. Since then, the company has deposited large amounts of industrial waste on the site next to the residences. Currently, the pile of steel waste reaches around 30 m in height. Furthermore, the condominium is located in a high-risk area, since levels of metals like zinc (Zn), nickel (Ni), copper (Cu), chromium (Cr), and lead (Pb) are also high, which reveals the extreme vulnerability of this population and the need for constant environmental monitoring [3].

The main routes of exposure to Cd are inhalation and ingestion, with approximately 25 to 60% of the element absorption occurring via the respiratory route. After absorption, the blood widely distributes the metal throughout the body, mainly to the liver and kidneys, with biological half-lives estimated at 4–19 and 6–38 years, respectively. About 80 to 90% of the metal present in the body is bound to metallothionein. Initially, the liver has high levels of Cd, but subsequently, hepatic levels reduce while their renal levels increase. After distribution, Cd is mainly eliminated in the urine, and its excretion increases with age [4].

There is evidence from in vitro and in vivo studies that Cd contamination may change the gene expression of various extracellular matrix (ECM) components, including matrix metalloproteinases (MMPs) [10,11,12,13,14,15]. MMPs are a family of zinc-dependent proteinases which degrade components of ECM, divided into classes according to substrate specificity: collagenases, stromelysin, elastases, and gelatinases [10]. Among the MMPs family, MMP-2 and MMP-9 stand out for their implication in multiple physiological and pathological processes, including tissue remodeling, inflammation-induced imbalance, infrastructure, and integrity of tissue barriers [11,13,14,15,16,17,18]. MMP2 is responsible for hydrolyzation of type IV collagen, a major structural component of the basement membrane, and is encoded by the *MMP2* gene (chromosome 16q13). *MMP9* (gene on chromosome 20q11.2-q13.1) facilitates vascular smooth muscle cell migration by acting as a proteolytic factor against type IV collagen [19]. Both *MMPs* genes are polymorphic, and their expression can vary according to the genotypic profile and, therefore, influence several health outcomes. Recently, it has been observed that single nucleotide polymorphisms (SNPs) in the *MMP-2* gene can modify the association between Cd exposure and hypertension in a Chinese population [18]. Thus, it is essential to understand the molecular mechanism of Cd toxicity and the individual susceptibility to health outcomes due to the presence of SNPs. The objectives of this study were to evaluate individuals chronically exposed to Cd regarding: (i) metal concentrations in blood and urine, (ii) frequencies of *MMP-2* and *MMP-9* SNPs and, (iii) to evaluate the influence of these SNPs on circulating levels of Cd in the human body.

## 2. Materials and Methods

### 2.1. Study Population and Clinical Evaluation

The Ethics Committee in Research from the National School of Public Health approved this study (CAAE number 40554820.9.0000.5240).

The condominium in Volta Redonda, Rio de Janeiro, Brazil has approximately 808 houses divided into 8 blocks with around 3000 residents, and all of these families live in a state of environmental injustice. The Volta Grande IV condominium is located in the Santo Agostinho neighborhood east of the city. A sample size was calculated, and 440 individuals were to be recruited. Residents were randomly selected through a drawing of the number of their houses, carried out in the presence of part of the population residing in the condominium. After the draw, those who attended the Primary Healthcare Center located in the condominium between April 2017 and October 2019 were invited to participate. The center was established to provide accessible, affordable, and available primary health care to people in accordance with the Alma Ata Declaration of 1978 by the member nations of the World Health Organization WHO. Primary care includes general practice, community pharmacy, dental, and optometry (eye health) services, among others. The inclusion criteria were adults over 18 years and living in the condominium for more than six months, and with the autonomy to answer the questionnaire. A convenience sample of 238 individuals agreed to participate in the research and signed the Free and Informed Consent Term.

Socio-demographic, environmental, and clinical information of the residents were taken from different questionnaires and validated by professionals from Fundação Oswaldo Cruz [3]. The clinical evaluation was performed by proper trained professionals, according to the Primary Healthcare Center routine protocols, and blood (*n* = 231) and urine (*n* = 203) samples were collected.

### 2.2. Cadmium Determination

Circulating levels of Cd were measured in blood (BCd) and urine (UCd) samples collected from the participants. The UCd was creatinine-corrected (UcCd). Blood specimens were collected in metal-free, heparinized vacutainer tubes, while urine was sampled directly in polyethylene containers previously tested for Cd contamination. The biological materials were transported to the laboratory under refrigeration and kept frozen until analysis. In the laboratory, the creatinine concentration in the urine was determined with a colorimetric kit from Doles^®^, using the spectrophotometric method with direct measurement by reaction with picric acid in alkaline medium, after deproteinization. Before measurement, the blood and urine samples were diluted in Triton X-100 and nitric acid, respectively. Two atomic absorption spectrometers, An Analyst 800 and 900, equipped with a transverse electrothermal atomizer, longitudinal Zeeman background corrector, AS-800 auto sampler, end cap pyrolytic graphite tubes, Lumina hollow cathode lamps, and all Perkin-Elmer, determined the concentration of BCd and UCd. The accuracy of the results was monitored by analyzing, in each series of samples, the following reference materials: Contox Heavy Metal Blood Control and Contox Metal Serum Control (Kaulson Laboratories, West Caldwell, NJ, USA); Lyphochek Urine Metals Control (Bio-Rad, Hercules, CA, USA) and Toxic Metals in Freeze-Dried Urine SRM 2670.

### 2.3. Polymorphisms Genotyping

DNA was extracted from blood samples using an extraction kit (Qiagen, Hilden, Germany) following the procedures recommended by the manufacturer. The genotyping analysis of *MMP-2* (rs7201 A > C and rs14070 C > T) and *MMP-9* (rs17576 A > G) were performed using a validated *TaqMan* allelic discrimination assay (C_3225976_10; C_11776087_1 and C_11655953_10, respectively). The TaqMan system for allelic discrimination consists of a set of primers and oligonucleotide probes designed specifically for each target SNP. The two probes are marked with different fluorescence, allowing the identification of the two possible alleles (rs7201 A or C, rs14070 C or T and rs17576 A or G) presents in the sample of the individuals. The fluorescence intensity is captured by the 7500 Real-Time System (Applied Biosystems, Foster City, CA, USA) equipment, discriminating the individuals’ genotypes: rs7201 AA, AC or CC; rs14070 CC, CT or TT; and rs17576 AA, AG or GG.

PCR amplification was performed in 8 μL reactions with 1 μL of template DNA (~10 ng/μL), 1 × TaqMan Universal Master Mix, 1 × each primer, and probe assay. Thermal cycling was initiated with a first denaturation step of 10 min at 95 °C, followed by 40 cycles of denaturation at 92 °C for 15 s and annealing at 60 °C for 1 min, as previously described [20]. Each reaction used two standardized positive controls of each polymorphism genotype to assure genotyping quality. Allelic and genotypic frequencies of the polymorphisms were directly determined by the 7500 Software v2.3 (Applied Biosystems, Foster City, CA, USA).

### 2.4. Data Analysis

Continuous variables were presented as mean, median, and their respective range. The distribution of Cd levels was tested by the Shapiro-Wilk normality test and the associations between continuous variables were evaluated by the Spearman’s linear correlation coefficient. Categorical variables were described as number (*n*) and percentages (%) and analyzed using the chi-square test or Fisher’s exact test, if necessary. The associations between categorical variables (sociodemographic, clinical, and genetic) and circulating levels of Cd were evaluated by determining the odds ratios (OR) and their respective 95% confidence intervals (95% CI), with adjustment for possible confounding factors, using binary logistic regression models. The adjustment model used was determined by the variables that had a significance level lower than or equal to 0.20 (*p* ≤ 0.20) in the univariate analysis, but which remained with a significance level of 0.05 (*p* ≤ 0.05) after model exit. Deviations from Hardy–Weinberg equilibrium (HWE) in *MMP-2* and *MMP-9* SNPs frequencies were assessed by the goodness-of-fit χ^2^ test, to investigate disruptions in genotype and allele frequencies in large populations due mutations, natural selection, nonrandom mating, genetic drift, and gene flow, also ruling out the possibility of misconducted genotyping assays.

All statistical analyzes were performed using the R Software (R Foundation for Statistical Computing, Vienna, Austria, version 4.2.1) and the Statistical Package for the Social Sciences (SPSS Inc., Chicago, IL, USA, version 20.0). A significance level of 0.05 was considered statistically significant.

## 3. Results

The present study comprised 238 individuals, of which 58% were female, and 50.8% were under 50 years old (mean 50.4 ± 14.8 years). Most of these individuals were non-smokers (69.3%) and non-drinkers (51.4%). The average time of residence in the condominium was 14.9 ± 5 years (median of 17 years), ranging from 6 months to 22 years, and with 41.8% of individuals residing for 17 years or more. The mean concentrations for BCd, UCd and UcCd were 0.70 ± 0.2 μg L^−1^ (median of 0.69 μg L^−1^, ranging from 0.28 μg L^−1^ to 1.62 μg L^−1^), 0.56 ± 0.55 μg L^−1^ (median of 0.35 μg L^−1^, ranging from 0.18 μg L^−1^ to 3.7 μg L^−1^), and 0.58 ± 0.63 μg g^−1^ (median of 0.35 μg g^−1^, ranging from 0.08 μg g^−1^ to 3.63 μg g^−1^), respectively. A correlation analysis between circulating levels of Cd and the independent variables age and time of residence showed that BCd and UcCd presented a linear association with age, with a Spearman’s linear correlation coefficient of 0.23 and 0.21, respectively (*p*-values = 0.001 and 0.006, respectively) (Data not shown). No significant correlations were found between blood and urine Cd concentrations. For subsequent analyses, BCd, UCd, and UcCd were stratified according to the medians obtained for each variable.

Table 1 displays the association between socio-demographic variables and Cd levels. Significant differences were observed only for BCd regarding the smoking status (*p*-value = 0.002), with the group smoker + former smoker presenting a higher chance of having BCd ≥ 0.70 μg L^−1^ (OR = 2.99; 95%CI = 1.55–5.85). UCd and UcCd levels were not significantly different considering sex, age, residence time, smoking, and drinking status groups.

Clinical anamnesis revealed respiratory diseases as the most prevalent NCD among residents of the condominium (28.9%), followed by cardiovascular (23.2%), renal (10.5%) and neurological diseases (8.8%), and neoplasms (4.4%). Considering the distribution of NCDs between the Cd levels, no significant differences were found (Data not shown in Table). However, renal diseases (*p*-value = 0.09) entered the binary logistic regression model for BCd because of the *p*-value ≤ 0.20 in the univariate analysis.

**Figure 1:**
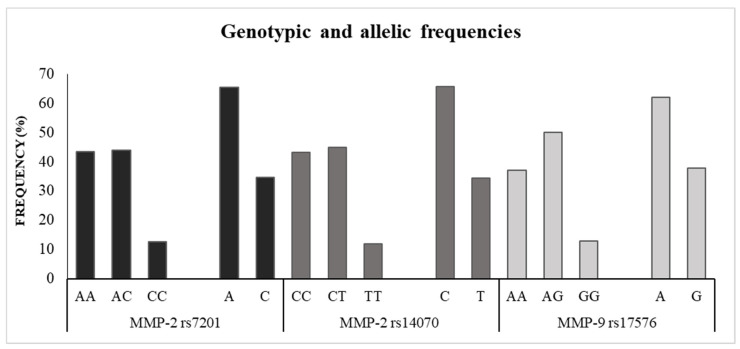
Genotypic and allelic frequencies of the MMP-2 and MMP-9 SNPs.

The rate of successful genotyping of the *MMP2 rs7201 A > C*, *MMP-2 rs14070 C > T* and *MMP-9 rs17576 A > G* SNPs were 99.1%, 98.7%, and 100%, respectively, and the distribution of all three SNPs was in HWE. As shown in Figure 1, the frequencies were 12.6%, 11.8%, and 12.9% for the variant genotypes *MMP2 rs7201 CC*, *MMP-2 rs14070 TT*, and *MMP-9 rs17576 GG*, while 34.6%, 34.3%, and 37.9% for the minor alleles *MMP2 rs7201 C*, *MMP-2 rs14070 T*, and *MMP-9 rs17576 G*. Table 2 presents the associations between the SNPs and circulating levels of Cd. The smoking status variable presented *p* < 0.05 in the binary logistic regression model for the BCd concentrations and, therefore, was used to calculate the adjusted OR. For the UCd and UcCd, no variable (sex or age) remained significant after leaving the model and, therefore, only the raw OR values were obtained. The circulating Cd levels were not significantly different regarding the *MMP-2 rs7201* and *rs14070 SNPs*. However, considering the *MMP-9 rs17576 A > G* SNP, the combination of heterozygous and homozygous variant genotypes (*AG + GG*) was associated with BCd ≥ 0.70 μg L^−1^ (OR = 2.11; 95%CI = 1.10–4.05) and UCd ≥ 0.56 μg L^−1^ (OR = 3.38; 95%CI = 1.82–7.65). Furthermore, considering only the *MMP-9 rs17576* alleles, there was also an association with the variant allele (*G*) and UCd ≥ 0.56 μg L^−1^ (OR = 1.72; 95%CI =1.12–2.65).

## 4. Discussion

The present study determined the Cd concentrations in blood and urine samples from residents of a condominium built over a steel slag disposal plot. Circulating levels of Cd were associated with smoking status and *MMP-9* rs17576 *A > G* SNP. The study population presented high prevalence of NCDs, mostly respiratory, cardiovascular, renal, and neurological diseases.

The average age of the participants, 50 years, was close to 56 years, found in a Chinese study that also investigated the association between *MMP* SNPs and Cd concentration from low dose environment exposure. No significant associations were found between Cd concentrations and *MMP* SNPs in the Chinese study, however, the *rs14070* and *rs7201* SNPs from *MMP2* gene were responsible for modifying the associations of urinary Cd with hypertension risk [18]. Likewise, the age group (18–86 years old) of the present study was similar to the range reported in a Canadian study (20–79 years old), which associated BCd and UCd with hypertension in an environmentally exposed population [9]. Both studies investigated the association between Cd concentrations and age, however neither found significant associations [9,18], such as the present study. Other studies with populations exposed to Cd also observed associations between higher Cd concentrations and age, sex, and current smoking [21,22]. In the present study, we also found an association between BCd ≥ 0.70 μg L^−1^ and smoking status, which is well described in the literature, as Cd is one of the main metals found in cigarettes and aside from the environmental exposure, cigarettes can also contribute to exposure to Cd and, therefore, to higher concentrations of this metal in the human body [23,24,25]. A review study from 2017 investigated the associations between blood, urine, and tissue Cd concentrations due to tobacco use and observed that Cd concentrations are often higher in smokers [24].

A study with 469 residents environmentally exposed to Cd in the Brazilian Amazon, showed a shorter exposure time (2–10 years) compared to the current study [23]. A recent study, published in 2021, investigated the association between heavy metal exposure and contributing factors in 814 residents living near a Zi smelter in Seokpo-myeon, South Korea. The study showed that participants living for ≥10 years in that area had higher UCd levels compared to those who had lived for less than 10 years [26]. In the present study, people have lived in the condominium and were exposed to Cd and other metals (e.g., Zn, Ni, Cu, Cr and Pb) for an average of 15 years, yet no association was found between circulating levels of Cd and residence time. Cd can be found in the plasma just a few days after exposure and accumulates in the fat tissue throughout a person’s lifetime [27], however, it is possible that other biological phenomena may influence this process. Considering that kidney function related to absorption and excretion of toxic metals is expected to change with increasing age, longer exposure such as the Korean study found (mean of 24 years), also means that people are older and, therefore, could present higher Cd levels due to impaired renal function [28]. This is worrisome since many condominium residents were over 50 years old and had already been exposed for more than 15 years of living on top of industrial waste. Besides that, the 30-m pile of steel waste near the condominium also contains residuals of other metals, characterizing a multiple exposure in which adverse effects are unknown [3,29].

The prevalence of NCDs in the study population was much higher than the prevalence in the Brazilian population [30]. Association between NCDs and Cd concentrations had already been observed in previous studies with increased risk of hypertension [18], atherosclerotic cardiovascular disease [31], neurological/neuropsychological [32], renal diseases [33], lung/respiratory disease [34] and cancer [33,35]. However, our results showed no association between NCD and Cd concentrations since it is a cross-sectional study, and is possible that the high prevalence of NCDs is due to an unmeasured variable in this population. The lack of association between NCDs and Cd may also result from a selection bias of the study population, which may not represent the true relationship observed in the entire population at risk. Although the sampling was initially carried out in a probabilistic way, not all selected individuals attended the Primary Healthcare Center or accepted to participate in the research. The refusal might be due to fear of retaliation, since many residents work or have relatives who work at the factory, which may have caused an underrepresentation of people with higher levels of Cd in the body and, probably, a higher prevalence of NCDs. In addition, the population currently residing in the condominium is mostly composed of adults, retirees, and the elderly.

It was also possible to determine the genotypic and allelic frequencies of the three *MMP* SNPs, with high rate of successful genotyping and within the HWE. All minor allele frequencies were in accordance with previous studies [18,36,37,38,39,40,41]. The *MMP-9* rs17576G allele frequency is 30.1% in the Chinese population (N = 1007) [40], 37.1% in the Romanian population (N = 197) [39], 21% in the Egyptian population (N = 100) [37], 36.1% in the Russian population (N = 347) [41] and 36.2% in the Slovenian population (N = 161) [38]. For the *MMP-2* rs7201C allele, the frequency is 24.6% in the Chinese population (N = 497) [18], 41.3% in the Slovenian population (N = 161) [38] and 45.4% in the German population (N = 143) [36]. Only one study describing the *MMP-2* rs14070T allele frequency was found, with 25.6% in the Chinese population (N = 497) [18]. Here, no associations were found for the *MMP-2* SNPs and Cd concentration. Wu and colleagues genotyped 7 SNPs in *MMP-2* and *MMP-9,* including the 2 *MMP-2* SNPs studied here. Only *MMP-2 rs7201 A > C* and *rs14070 C > T* were positively associated with increased urinary Cd concentration and hypertension risk in Chinese population [18]. In the Brazilian population of the present study, the *MMP-9 rs17576 A > G* SNP was associated with a higher chance of having BCd ≥ 0.70 μg L^−1^ and UCd ≥ 0.57 μg L^−1^. The *MMP-9 rs17576 A > G* SNP is a nonsynonymous alteration that may change protein function or structure, also influencing splicing [38]. Therefore, the presence of the *rs17576 G* allele causes a reduction in MMP9 expression, in comparison to the wild allele *rs17576 A* [42]. There are still no studies evaluating the association between the expression of *MMP2* and *MMP9* and circulating levels of cadmium in the human body. Nevertheless, studies have evaluated this association in vitro and in experimental models, which can be a starting point for more robust studies that consider the complexity of the human organism as a whole. Several studies describe that treatment with Cd increases *MMP2* and *MMP9* expression in vitro [11,13,15,43] and in experimental models [44]. It is known that MMPs might mitigate the processes induced by exposure to heavy metals, such as inflammation and oxidative stress [18,45] Several studies have correlated the MMP profiles with other heavy metals like Pb, arsenic, and mercury [46,47]. Although data about these SNPs are scarce, we hypothesized that, given the presence of a SNP that reduces MMP9 expression, it increases the likelihood of negative effects associated with Cd exposure as happens with other heavy metals.

Our results highlight the necessity of constant monitoring of the individuals from the Volta Redonda condominium, as they present a basal environmental exposure that can be very harmful. In addition, this is the first study to evaluate the influence of *MMP-2 rs7201 A > C* and *MMP-9 rs17576 A > G* SNPs on the circulating levels of Cd in the Brazilian population. Nevertheless, some limitations also need to be highlighted, such as limited financial resources for genetic analysis, the selection bias resulted from refusal to participate in the study, the non-measurement of known confounding factors such as other genes/SNPs, blood/serum Zn/Cd ratio, medications in use, diet, seasonal changes, concentration of others metals, use of vape devices, occupational exposures, and traffic related Cd exposure that may contribute to contaminated soil ingestion and dust inhalation.

## 5. Conclusions

In summary, smoking status and the *MMP-9* rs17576 A > G SNP were associated with higher chance (approximately 3×) of having higher Cd concentration. These results may be used to construct a broad database from different populations to identify the effects of the environmental exposure to heavy metals on the health status of individuals. The knowledge regarding possible individual predisposing factors is essential to understand the molecular mechanism of toxicity of Cd, in addition to making public policies to mitigate the negative impact of industrial waste and to improve protocols for screening, prevention and monitoring of the health situation of populations living in soil contaminated by steel industry waste.

## Figures and Tables

**Table 1:** Association between socio-demographic variables and Cd concentrations.

Characteristics	BCd ^a,b^ (*n* = 231)		Ucd ^a,c^ (*n* = 203)		UcCd ^a,d^ (*n* = 189)	
	≤0.70	>0.70	≤0.56	>0.56	≤0.58	>0.58
**Sex**	*n* (%)					
Women	74 (56.9)	62 (61.4)	85 (61.6)	33 (50.8)	80 (60.2)	32 (57.1)
Men	56 (43.1)	39 (38.6)	53 (38.4)	32 (49.2)	53 (39.8)	24 (42.9)
**Age (years) ^g^**	*n* (%)					
≤50	60 (55.0)	41 (46.1)	60 (50.4)	27 (44.3)	62 (52.5)	22 (40.0)
>50	49 (45.0)	48 (53.9)	59(49.6)	34 (55.7)	56 (47.5)	33 (60.0)
**Smoking Status ^f^**	*n* (%)					
Non-smoker	77 (80.2)	46 (57.5)	78 (69.0)	36 (72.0)	79 (73.1)	30 (63.8)
Smoker ^e^	19 (19.8)	34 (42.5)	35 (31.0)	14 (28.0)	29 (26.9)	17 (36.2)
**Drinking Status**	*n* (%)					
Non-drinker	49 (50.0)	43 (53.8)	60 (52.6)	24 (47.1)	56 (51.4)	26 (54.2)
Drinker ^e^	49 (50.0)	37 (46.2)	54 (47.4)	27 (52.9)	53 (48.6)	22 (45.8)
**Residence Time (years) ^g^**	*n* (%)					
≤17	60 (57.1)	44 (60.3)	57 (55.3)	36 (61.0)	63 (60.6)	28 (53.8)
>17	45 (42.9)	29 (39.7)	46 (44.7)	23 (39.0)	41 (39.4)	24 (46.2)

^a^ Cd levels in µg L^−1^. ^b^ Missing information of 33 individuals for age, 55 for smoking status and 53 for drinking status and residence time. ^c^ Missing information of 23 individuals for age, 40 for smoking status, 38 for drinking status and 41 for residence time. ^d^ Missing information of 16 individuals for age, 34 for smoking status, 32 for drinking status and 33 for residence time. ^e^ Smoker and drinker groups included current smoker/drinker and former smoker/drinker. ^f^ *p*-value ≤ 0.05 for BCd. *p*-value obtained through the Chi-squared Test (Pearson *p*-value) or Fisher’s exact test. ^g^ Age and residence time groups were defined according to the median values.

**Table 2:** Association between polymorphisms and Cd concentrations.

SNPs	BCd ^a^ (*n* = 231)		Ucd ^a^ (*n* = 203)		UcCd ^a^ (*n* = 189)	
	≤0.70	>0.70	≤0.56	>0.56	≤0.58	>0.58
***MMP9 A > G (rs17576) ^b^***	*n* (%)					
AA	55 (42.6)	30 (30.9)	62 (46.3)	13 (20.3)	55 (42.3)	17 (30.9)
AG	56 (43.4)	56 (57.8)	55 (41.0)	43 (67.2)	54 (41.5)	33 (60.0)
GG	18 (14.0)	11 (11.3)	17 (12.7)	8 (12.5)	21 (16.2)	5 (9.1)
AG + GG ^e^	74 (57.4)	67 (69.1)	72 (53.7)	51 (79.7)	75 (57.7)	38 (69.1)
A	166 (64.3)	116 (59.8)	179 (66.8)	69 (53.9)	164 (63.1)	67 (60.9)
G ^f^	92 (35.7)	78 (40.2)	89 (33.2)	59 (46.1)	96 (36.9)	43 (39.1)
***MMP2 A > C (rs7201) ^c^***	*n* (%)					
AA	61 (47.3)	38 (39.6)	65 (48.9)	26 (40.6)	61 (47.3)	27 (49.1)
AC	52 (40.3)	45 (46.9)	49 (36.8)	31 (48.5)	51 (39.5)	21 (38.2)
CC	16 (12.4)	13 (13.5)	19 (14.3)	7 (10.9)	17 (13.2)	7 (12.7)
AC + CC	68 (52.7)	58 (60.4)	68 (51.1)	38 (59.4)	68 (52.7)	28 (50.9)
A	174 (67.4)	121 (63.0)	179 (67.3)	83 (64.8)	173 (67.1)	75 (68.2)
C	84 (32.6)	71 (37.0)	87 (32.7)	45 (35.2)	85 (32.9)	35 (31.8)
***MMP2 C > T (rs14070) ^d^***	*n* (%)					
CC	60 (46.9)	39 (40.6)	65 (48.9)	26 (41.3)	61 (47.3)	27 (50.0)
CT	53 (41.4)	46 (47.9)	51 (38.3)	30 (47.6)	52 (40.3)	20 (37.0)
TT	15 (11.7)	11 (11.5)	17 (12.8)	7 (11.1)	16 (12.4)	7 (13.0)
CT + TT	68 (53.1)	57 (59.4)	68 (51.1)	37 (58.7)	68 (52.7)	27 (50.0)
C	173 (67.6)	124 (64.6)	181 (68.0)	82 (65.1)	174 (67.4)	74 (68.5)
T	83 (32.4)	68 (35.4)	85 (32.0)	44 (34.9)	84 (32.6)	34 (31.5)

^a^ Cd levels in µg L^−1^. ^b^ Missing information of 5 individuals for BCd, 5 individuals for UCd and 4 individuals for UcCd. ^c^ Missing information of 6 individuals for BCd, 6 individuals for UCd and 5 individuals for UcCd. ^d^ Missing information of 7 individuals for BCd, 7 individuals for UCd and 6 individuals for UcCd. ^e^ *p*-value ≤ 0.05 for UCd and *p*-value = 0.03 (adjusted) for BCd in comparison with the wild genotype (*AA*). ^f^ *p*-value ≤ 0.05 for UCd in comparison with the wild allele (*A*).

## Data Availability

Data sharing not applicable.
